# Feasibility of a Transcutaneous Tibial Nerve Stimulation Device Use in Overactive Bladder Patients: A Pilot Study From a Single Tertiary Care Center

**DOI:** 10.3389/fneur.2022.872200

**Published:** 2022-04-13

**Authors:** Xunhua Li, Xing Li, Zhonghan Zhou, Hui Zhao, Limin Liao

**Affiliations:** ^1^Department of Urology, China Rehabilitation Research Center, School of Rehabilitation, Capital Medical University, Beijing, China; ^2^University of Health and Rehabilitation Sciences, Qingdao, China; ^3^Cheeloo College of Medicine, Shandong University, Jinan, China

**Keywords:** transcutaneous tibial nerve stimulation, overactive bladder, effectiveness, clinical, urodynamic parameters

## Abstract

**Purpose:**

To evaluate the preliminary efficacy, safety, and acceptability of a transcutaneous tibial nerve stimulation (TTNS) device in overactive bladder (OAB) patients.

**Methods:**

Twenty OAB patients who failed with conservative treatments were recruited consecutively. All patients received 60 min of daily unilateral stimulation for 4 weeks using a smart wearable transcutaneous tibial nerve stimulation device and the stimulations were at 20 Hz frequency, 200 μs pulse width. OAB symptoms were observed at baseline and week 4, using a 3-days voiding diary, the overactive bladder symptom score (OABSS), the perception of bladder condition (PPBC), and the American Urological Association Symptom Index Quality of Life Score (AUA-SI-QoL). Urodynamic characteristics were measured to determine the pilot efficacy of the device during the treatment comparing the baseline parameters to the post-treatment parameters.

**Results:**

Among the patients, 15 cases were OAB-dry and five cases were OAB-wet. All patients were evaluated at the end of the study and no significant side effects were found during the treatment. The daily micturition frequency and the number of incontinence episodes per day were reduced from 15.10 ± 1.61 to 12.00 ± 4.56, and 3.20 ± 0.80 to 0.47 ± 0.38, respectively. The mean voiding volume was increased from 130.10 ± 53.07 to 157.30 ± 66.95 mL. The OABSS, AUA-SI-QoL, and PPBC were reduced from 9.35 ± 1.39 to 5.9 ± 2.36, 5.70 ± 0.47 to 3.85 ± 1.04, and 5.70 ± 0.47 to 4.35 ± 0.86, respectively. The first sensation of bladder filling (1st SBF), maximal bladder capacity (MBC), and mean compliance were increased from 87.50 (60.00–167.50) to 150.00 (104.00–211.30) mL, 175.00 (120.30–354.00) to 255.00 (151.50–491.50) mL, and 36.67 (12.44–39.69) to 40.00 (20.00–52.50) mL/cmH_2_O, respectively. The maximum detrusor pressure (Pdet. max) was reduced from 14.50 (5.00–35.25) to 11.00 (6.00–20.00) cmH_2_O.

**Conclusion:**

The preliminary results demonstrated that the TTNS device was safe, effective, and acceptable to use in OAB patients, but the results need to be substantiated by conducting more randomized controlled studies further.

## Introduction

Overactive bladder (OAB) is defined as “urinary urgency, usually with frequency and nocturia, with or without urgency urinary incontinence” by the International Continence Society (ICS) (1). Usually, OAB is considered a syndrome in the absence of urinary tract infection or other pathological conditions. It is highly prevalent, affecting up to 12% of the adult population, and has a significant negative impact on the quality of life (2–4). OAB is categorized into OAB-wet and OAB-dry OAB based on the presence or absence of urinary incontinence. Women are more likely affected, especially by OAB-wet, and the incidence increases with advancing age (5).

Traditional and contemporary medical treatments are used in managing OAB. Antimuscarinic and β-adrenergic agents are frequently used pharmacological drugs in OAB patients. Invasive therapies benefit patients with refractory symptoms who show no improvement with conservative and pharmacological interventions, such as OnabotulinumtoxinA (BoNT-A) injection, sacral neuromodulation (SNM), and percutaneous tibial nerve stimulation (PTNS). The BoNT-A was approved in the treatment of urinary incontinence, secondary to neurogenic detrusor overactivity in 2011 (6) and used in the treatment of OAB in 2013 by the US Food and Drug Administration (FDA) (4). The BoNT-A administration is performed with the guidance of a cystoscope under intravenous sedation, and it is a minimally invasive procedure. However, reinjection every 6 months may be needed because of the self-limited duration of its action (7). The SNM received FDA approval to be adopted in urge incontinence in 1997 and urgent, frequent, and non-obstructive urinary retention in 1999 (8). However, an experimental period is needed to assess the effectiveness before implanting a permanent pulse generator. The SNM is a minimally invasive treatment for OAB, but the economic cost is a big challenge for some patients. Tibial nerve stimulation (TNS) is another option for OAB patients who are refractory to conservative therapies (9). At present, three types of TNS methods are adopted: percutaneous tibial nerve stimulation (PTNS), implantable tibial nerve stimulation (ITNS), and transcutaneous tibial nerve stimulation (TTNS) (10). The tip of a needle electrode is inserted into the medial malleolus during PTNS, which also brings a series of complications such as bleeding, infection, and pain. In the ITNS procedure, a stimulator needs to be implanted into the tibial nerve, which induces similar side effects as in the PTNS procedure. In addition, both PTNS and ITNS need to be performed by professional medical workers. In this study, we aimed to evaluate the preliminary efficacy, safety, and impact of a TTNS device in patients.

## Materials and Methods

### Study Design and Participants

This was a single-center self-controlled pilot study. Patients who had refractory OAB and failed with conservative treatment were recruited from our department from October 2020 to October 2021. The study was approved by the institutional review board. The inclusion criteria were as follows: patients with the age from 18 to 75 years, patients who agreed to voluntary participation in this clinical trial and provided consent to TTNS treatment, and patients who maintained a 3-day urinary diary with an average of ≥8 voids per 24 h and a 7-day washout period for the treatment with anticholinergic drugs and β3 adrenergic receptor agonists before the TTNS sessions. Otherwise, the use of drugs was unchanged during the treatment period. The exclusion criteria were as follows: patients who had uncontrolled symptomatic urinary tract infection, bladder tumor, urinary stones, pregnancy, a pacemaker or implantable defibrillators during the therapy, combined renal insufficiency (blood creatinine; >1.5 times the upper limit of normal), epilepsy, Alzheimer's disease, cerebral atrophy, acute stage of cerebrovascular disease, cognitive impairment, Parkinson's disease, complete spinal cord injury, mental illnesses leading to lack of cooperation with doctors, skin lesions at the treatment site, grade III or higher pelvic organ prolapse, other concomitant diseases that affect the efficacy of the trial, residual urine volume of >100 mL, indwelling urinary catheter or intermittent catheterization, nursing, plan to conceive during the study period, or participation in other drug or device clinical trials within 1 month prior to enrollment.

### TTNS Device

**Figure 1:**
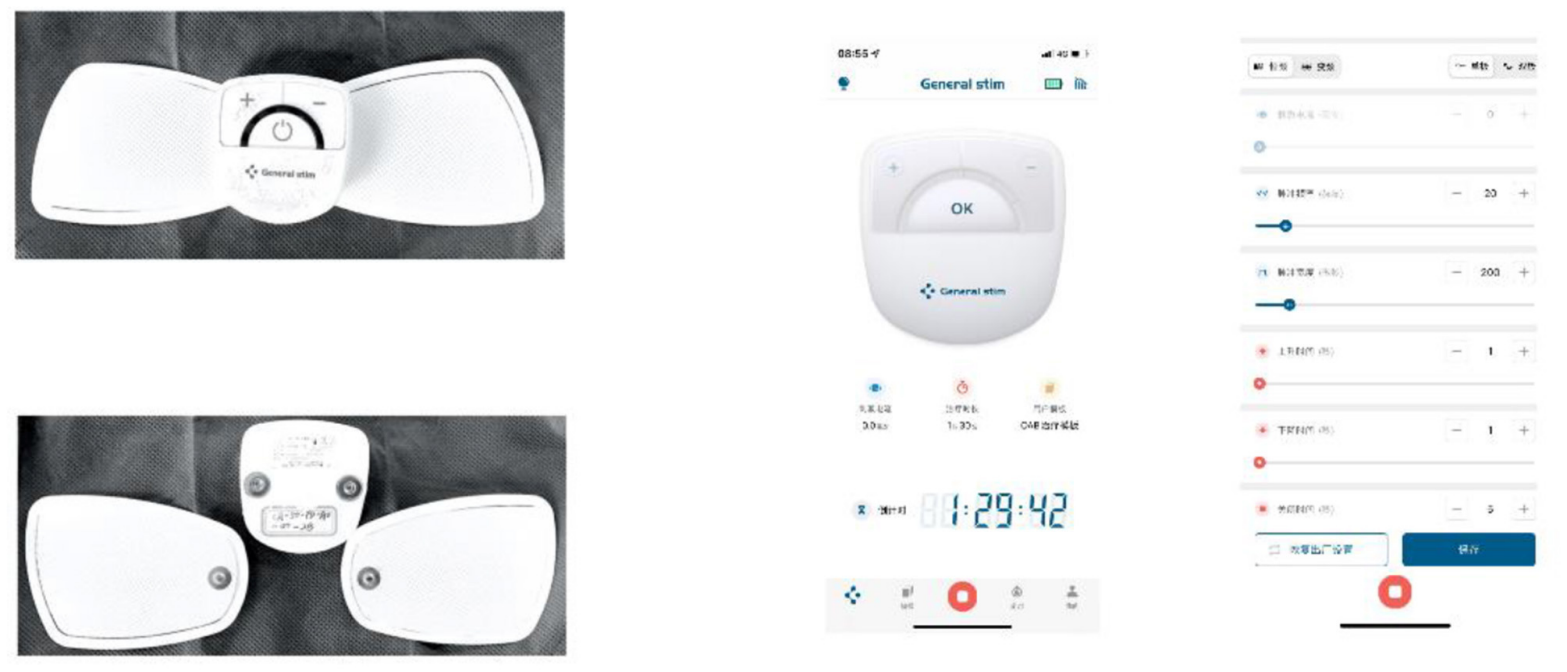
TTNS device (General Stim Inc., Hangzhou, Zhejiang, China) and the mobile terminal unit.

The butterfly shape TTNS device (General Stim Inc., Hangzhou, Zhejiang, China; Figure 1), consisted of three parts: the stimulation unit, surface electrodes, and the mobile terminal unit. The dimension of the stimulation unit with a rechargeable lithium battery was 58.3 ^*^ 55.6 ^*^ 12.8 mm^3^ (width, height, and depth), and the weight was only 70 g. With two working modes, program-controlled mode and offline mode, the stimulator was applied to patients regardless of the presence of an intelligent terminal. But more stimulation parameters were modulated under the program-controlled mode, thus patients could adjust the stimulation parameters on the terminal device by themselves or under the instruction of doctors. The ranges for stimulation parameters were as follows: 0–30 mA intensity; 1–200 Hz frequency; and 100–1,000 μs pulse width. The electrodes are in the shape of butterfly wings, about 86 mm long, wide outside and narrow inside, with a width of about 55 mm on the outside and a button on the inside to connect to the stimulator. The adhesive electrodes were removable and replaceable, and the surface was made of conductive silicone for comfortable use. The program control software in the mobile terminal was connected with the stimulator by Bluetooth. Meanwhile, it was able to export the treatment history with different formats: trend, bar, and pie charts with a more visual reference for the follow-up by the doctor.

### TTNS Procedure

**Figure 2:**
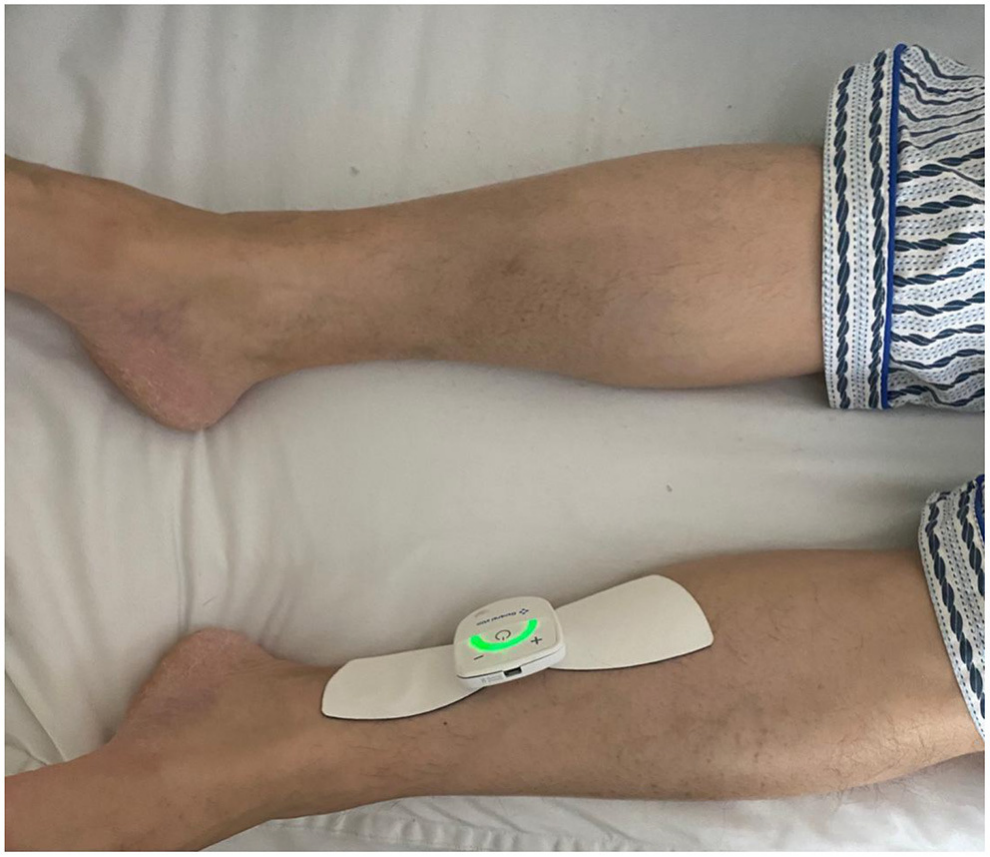
Placement of the stimulation electrode.

After wiping the skin of the electrode covered area on the body with saline cotton balls, the stimulator with two gel-based electrodes was placed ~3 fingers above the medial malleolus along the tibial nerve alignment (Figure 2). Patients underwent a 5-min test stimulation to ensure tolerability. The TTNS device settings (20 Hz frequency and 0.2 ms pulse width) were based on the previous study (11, 12). The only variable parameter was the stimulatory current (0–30 mA) that was increased slowly to a maximal intensity, which was comfortable to the patients with the observable involuntary toe contractions. After the 5-min test trial, patients who were unable to continue stimulation due to discomfort or anxiety were excluded from the study. The stimulation was performed 1 h daily lasting for 4 weeks.

### Clinical and Urodynamic Evaluation

Patients completed two validated questionnaires 3-days before and after the TTNS sessions, including a 3-days voiding diary, overactive bladder symptom score (OABSS), American Urological Association Symptom Index Quality of Life Score (AUA-SI-QOL), and perception of bladder condition (PPBC). The micturition daily frequency, mean voiding volume, and the number of daily incontinence episodes were evaluated from the 3-days voiding diary. To evaluate the efficacy of the TTNS device in acute stimulation, the urodynamic examination was performed before the TTNS treatment. Another examination was performed during the procedure of TTNS when the first urodynamics evaluation was completed. All urodynamic measurements were taken according to Good Urodynamic Practices (13). The following urodynamic evaluation parameters were used: the first sensation of bladder filling (1st SBF), maximal bladder capacity (MBC), the maximum detrusor pressure (Pdet. Max), and mean compliance. Any adverse effect by the device was recorded during the treatment.

### Statistical Analysis

Statistical analyses were performed using GraphPad Prism software (version 9.1.1, GraphPad Software, La Jolla, CA, USA). The Kolmogorov-Smirnov test was used to verify normal distribution. Descriptive data were reported as mean ± SD or median (25–75%) according to the normality of the data. Student's *t*-test or Wilcoxon test was performed for paired continuous variables depending on the type of distribution. *P*-value of < 0.05 was considered significant.

## Results

**Figure 3:**
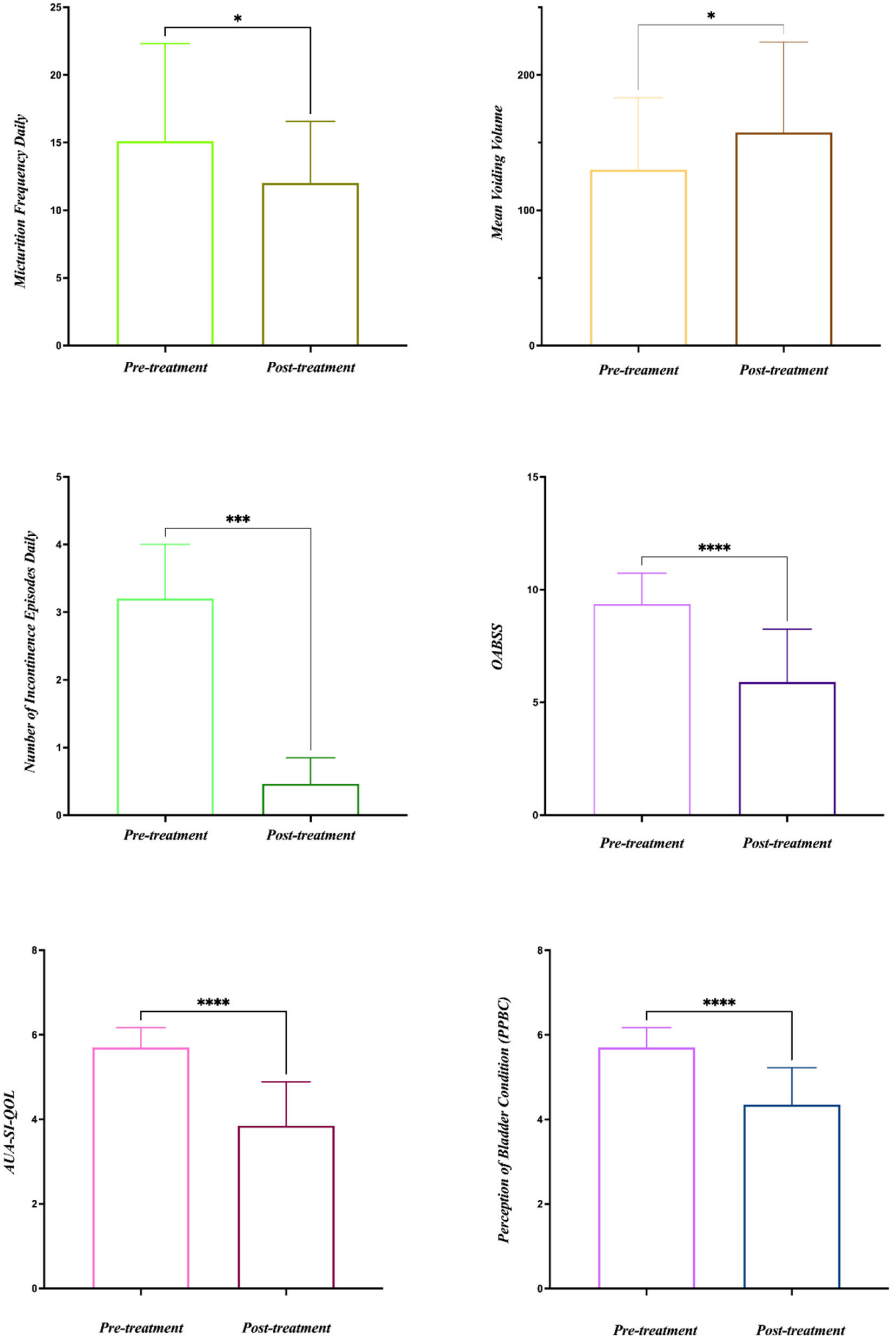
Clinical parameters pre- and post- treatment. *P < 0.05; ***P < 0.001; ****P < 0.0001.

**Figure 4:**
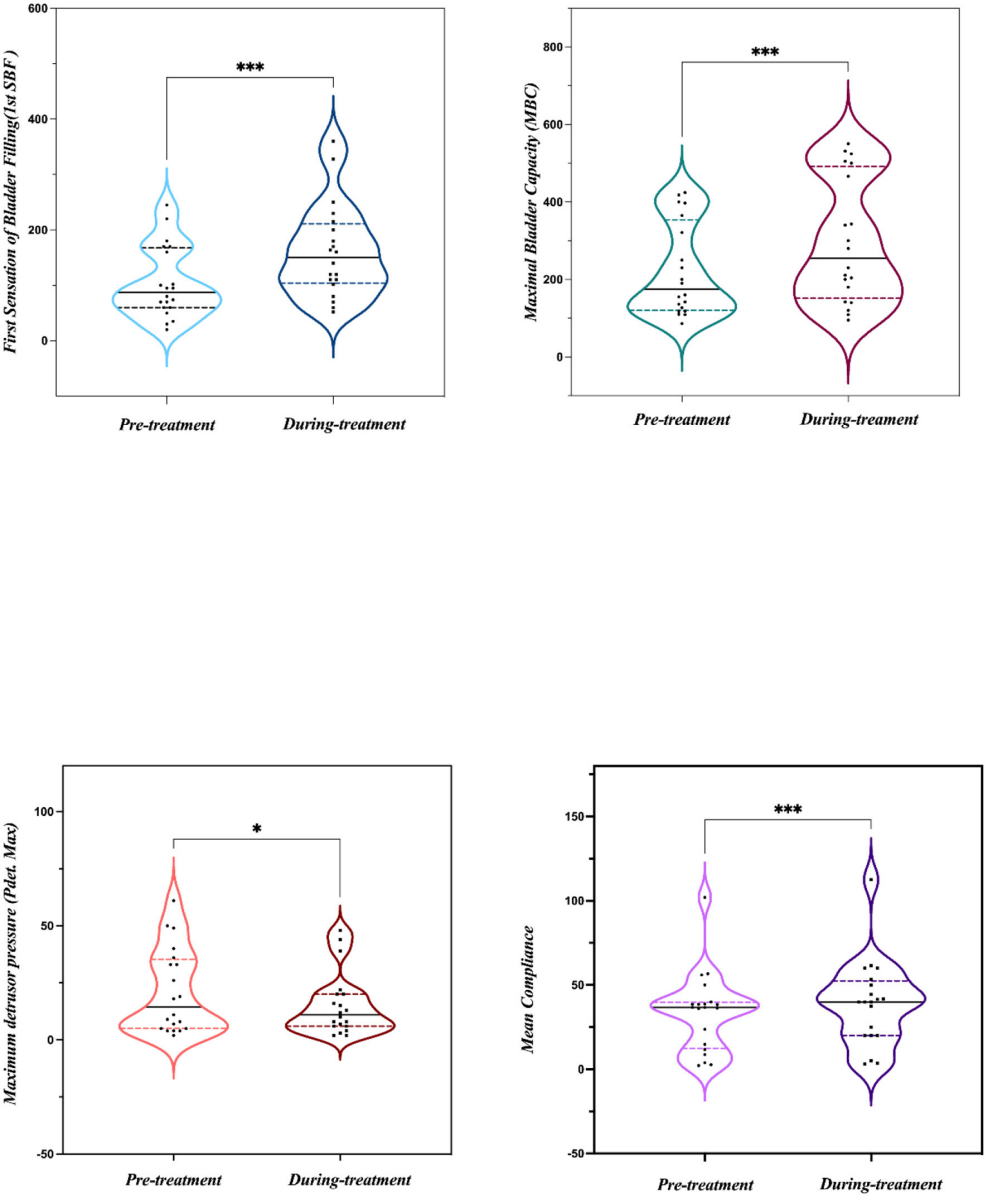
Urodynamic parameters before and during the treatment. *P < 0.05; ***P < 0.001.

**Figure 5:**
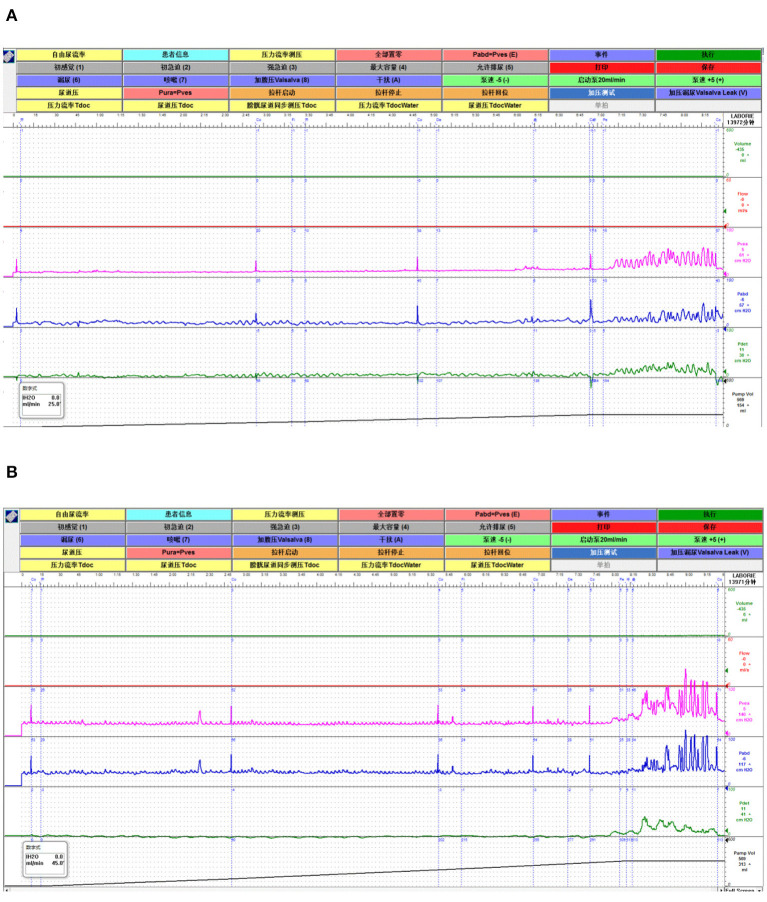
A representative of the urodynamic images pre- and during TTNS. **(A)** Pre-stimulation. **(B)** During-stimulation.

All 20 patients were evaluated at the end of the study and no significant side effects were found during the treatment. The baseline data are shown in Table 1. Among the 20 patients, five patients had OAB-wet, and the daily incontinence time was significantly reduced after the TTNS treatment. The clinical (Figure 3) and urodynamic (Figures 4, 5) parameters were significantly improved at varying degrees compared to pre-treatment values (Table 2). The micturition daily frequency and number of incontinence episodes per day were reduced from 15.10 ± 1.61 to 12.00 ± 4.56, and 3.20 ± 0.80 to 0.47 ± 0.38, respectively. The mean voiding volume was increased from 130.10 ± 53.07 mL to 157.30 ± 66.95 mL. The OABSS, AUA-SI-QOL, and PPBC were reduced from 9.35 ± 1.39 to 5.9 ± 2.36, 5.70 ± 0.47 to 3.85 ± 1.04, and 5.70 ± 0.47 to 4.35± 0.86, respectively. The 1st SBF, MBC, and mean compliance were increased from 87.50 (60.00–167.50) to 150.00 (104.00–211.30) mL, 175.00 (120.30–354.00) to 255.00 (151.50–491.50) mL, and 36.67 (12.44–39.69) to 40.00 (20.00–52.50) mL/cmH_2_O, respectively. The Pdet. Max value was reduced from 14.50 (5.00–35.25) to 11.00 (6.00–20.00) cmH_2_O. When the OAB-dry and OAB-wet subgroups were analyzed separately, some of the results did not achieve statistical significance because of the small sample size. In OAB-dry group, the micturition daily frequency was reduced from 13.00 (10.67–16.67) to 11.33 (9.33–15), The mean voiding volume was increased from 131.60 ± 55.97 to 162.10 ± 74.99 mL. The OABSS, AUA-SI-QOL, and PPBC were reduced from 9.27 ± 1.58 to 6.20 ± 2.65, 6.00 (5.00–6.00) to 4.00 (4.00–5.00), and 6.00 (5.00–6.00) to 5.00 (4.00–5.00), respectively. The 1st SBF, MBC, and mean compliance were increased from 105.80 ± 61.05 to 134.2 ± 58.85 mL, 218.00 ± 119.40 to 272.5 ± 146.60 mL, and 36.54 ± 25.15 to 41.66 ± 27.25 mL/cmH_2_O, respectively. In the OAB-wet group, the micturition daily frequency was significantly reduced from 14.33 ± 5.60 to 11.73 ± 5.00. However, no significant difference was detected between the change of other parameters. The improvements between OAB-dry and OAB-wet after the treatment of TTNS were also evaluated, and the results showed that there were no significantly difference between them in both clinical and urodynamic parameters (*p* > 0.05) but the first sensation of bladder filling which was increased from −28.40 ± 33.00 to −141.8 ± 94.27 (*p* < 0.05) (Table 3).

**Table 1:** Baseline demographic and clinical characteristics.

	**IQR/Mean ±SD/n (%)**
**Gender**	
Female	12 (60%)
Male	8 (40%)
Age, years	33.50 (29.00–59.75)
BMI, kg/m^2^	22.96 (19.98–24.49)
**OAB Type**	
OAB-Dry	15 (75%)
OAB-Wet	5 (25%)
Duration of OAB symptoms, years	3.35 ± 1.54

*SD, standard deviation; IQR, interquartile range*.

**Table 2:** Clinical and urodynamic parameters before and at completion of PTNS treatment.

	**Parameters**	**Pre-treatment**	**Post-treatment**	***P*-value**
Clinical parameters	Micturition Frequency Daily	15.10 ± 1.61	12.00 ± 4.56	0.0119
	Mean Voiding Volume (mL)	130.10 ± 53.07	157.30 ± 66.95	0.0131
	Number of Incontinence Episodes per Day	3.20 ± 0.80	0.47 ± 0.38	0.0004
	OABSS	9.35 ± 1.39	5.9 ± 2.36	<0.0001
	AUA-SI-QOL	5.70 ± 0.47	3.85 ± 1.04	<0.0001
	PPBC	5.70 ± 0.47	4.35 ± 0.86	<0.0001
	**Parameters**	**Pre-treatment**	**During-treatment**	***P*** **-value**
Urodynamic parameters	1st SBF (mL)	87.50 (60.00–167.50)	150.00 (104.00–211.30)	0.0001
	MBC (mL)	175.00 (120.30–354.00)	255.00 (151.50–491.50)	0.0001
	Pdet.Max (cmH_2_O)	14.50 (5.00–35.25)	11.00 (6.00–20.00)	0.0184
	Mean compliance (mL/cmH_2_O)	36.67 (12.44–39.69)	40.00 (20.00–52.50)	0.0003

**Table 3:** The comparison between the OAB-dry and OAB-wet subgroups in both clinical and urodynamic parameters.

	**OAB-dry**			**OAB-wet**			**Improvement**		
	**Pre-**	**Post-**	***P***	**Pre-**	**Post-**	***P***	**OAB-dry (pre-post)**	**OAB-wet (pre-post)**	***P***
Micturition frequency	13.00 (10.67–16.67)	11.33 (9.33–15.00)	0.004	14.33 ± 5.60	11.73 ± 5.00	0.035	2.67 (0.66–3.67)	3.34 (0.67–4.17)	0.655
Voiding volume (mL)	131.60 ± 55.97	162.10 ± 74.99	0.031	100.90 (93.95–169.20)	133.80 (121.00–170.00)	0.313	−30.44 ± 49.31	−17.73 ± 21.77	0.589
OABSS	9.27 ± 1.58	6.20 ± 2.65	<0.001	10.00 (9.00–10.00)	5.00 (4.50–5.50)	0.063	4.00 (1.00–5.00)	5.00 (3.50–5.50)	0.266
PPBC	6.00 (5.00–6.00)	5.00 (4.00–5.00)	0.001	6.00 (5.00–6.00)	4.00 (3.50–4.00)	0.063	1.00 (0.00–2.00)	2.00 (1.50–2.00)	0.216
Qol	6.00 (5.00–6.00)	4.00 (4.00–5.00)	<0.001	6.00 (5.00–6.00)	3.00 (3.00–3.50)	0.063	2.00 (1.00–3.00)	3.00 (1.50–3.00)	0.222
1st SBF (mL)	105.80 ± 61.05	134.20 ± 58.85	0.005	70.00 (55.00–59.50)	230.00 (145.00–344.00)	0.063	−28.40 ± 33.00	−141.80 ± 94.27	<0.001
MBC (mL)	218.00 ± 119.40	272.5 ± 146.60	0.002	237.20 ± 132.10	376.40 ± 191.10	0.077	−54.53 ± 56.44	−139.20 ± 131.10	0.054
Pdet.Max (cmH_2_O)	9.00(5.00–33.00)	10 (6.00–22.00)	0.059	23.40 ± 15.58	11.40 ± 5.98	0.163	3.93 ± 7.51	12.00 ± 15.73	0.134
Compliance (mL/cmH_2_O)	36.54 ± 25.15	41.66 ± 27.25	0.014	24.81 ± 17.04	30.85 ± 18.10	0.058	−5.12 ± 7.09	−6.04 ± 5.13	0.794

## Discussion

OAB syndrome significantly affects the quality of life and causes a huge economic burden for managing the condition (14, 15). As the third line of treatment, neuromodulation is found to be an effective therapy for the OAB patients either in an open- or a closed-loop model of treatment (16, 17).

Several studies reported the efficacy of TTNS in treating OAB. A prospective observational study (18) showed its effectiveness in one-half of the patients studied after they failed conservative and pharmacological treatments. Leroux et al. (19) confirmed the efficacy of TTNS in OAB patients. The result showed that TTNS was successful after 3 months of treatment in 71% of patients and the mean urinary symptom profile score remained significantly lower than the baseline value until 12 months after treatment. Araujo et al. (20) conducted a prospective, randomized, double-blinded, sham-controlled clinical trial and found that the stimulation group showed a reduction in night-time urinary frequency, urinary urgency, urgency incontinence episodes, use of pads, and OAB-V8 and King's Health Questionnaire scores. In a 30- and 90-day follow-up, 53.3 and 33.3% of patients who underwent stimulation, reported complete symptom relief after discontinuation of the intervention, respectively. Patients who underwent stimulation showed a statistically significant improvement of symptoms as compared with the sham patients. Also, a randomized, active-controlled clinical trial (21) evaluated the effectiveness of TTNS compared to PTNS in OAB patients who responded to an initial 12-week course of PTNS. The result showed that urinary frequency, episodes of urinary urgency, and episodes of urge urinary incontinence were significantly improved and no significant difference between the two groups was found, demonstrating that TTNS was effective in maintaining the symptom improvement in OAB patients as PTNS.

As an effective and less invasive method, TNS plays an important role in treating OAB. However, PTNS needs a needle electrode to be inserted into the medial malleolus, causing difficulty in conducting close-looped treatment. ITNS needs to be implanted into the tibial nerve that brings the complications such as bleeding, infection, and pain. However, there are no specific TTNS devices that have been approved for the treatment of OAB until now. The GEKO (22), which has been used by some researchers in the treatment of OAB, was approved for the prevention of deep vein thrombosis. Although the GEKO device has many benefits, it could not connect to the intelligent terminals of the internet of every era. With the benefits of multiple stimulation parameters being adjusted by patients or doctors, the wearable TTNS device (General Stim Inc., Hangzhou, Zhejiang, China) also can be connected to intelligent terminals such as smartphones by Bluetooth, and the stimulator can be modulated by the intelligent terminal itself if the terminal receives signals from the human body. Simultaneously, the device is aesthetic and not traumatic from using adhesive electrodes. Patients can use the stimulator at home and modulate the stimulation parameters themselves from the instruction of doctors. It can avoid frequent hospital visits for OAB patients, especially during this COVID-19 pandemic.

Before this study, an animal experiment using nine male cats was conducted to test the efficacy of the device on bladder reflex. Two self-adhesive electrodes of the TTNS device were placed at the left leg and ITNS was applied to stimulate the tibial nerve of the right leg. The result showed that TTNS at four times threshold, six times threshold, and the maximum current intensity of 24 mA significantly increased the bladder capacity compared to the control level of the output current. The inhibitory effects of TTNS and ITNS had no significant difference (unpublished data).

This study revealed that both the daily micturition frequency and the number of incontinence episodes per day were significantly reduced and the mean voiding volume increased significantly after 1 month of TTNS. The preliminary results showed the efficacy of the TTNS device for long time stimulation. The immediate efficacy of the device was also supported by the improvement of urodynamic parameters, including 1st SBF, MBC, Pdet. Max, and mean compliance. Even though when the OAB-dry and OAB-wet subgroups were analyzed separately, some of the results did not achieve statistical significance because of the small sample size, the *p*-value of many parameters achieved marginal significance (Table 3). The device was also found to be safe since no significant adverse effects were noted.

This study had some limitations, and these limitations should be overcome in future elucidations. First, this was a pilot study, and the sample size of this study was not large enough and only 20 subjects were recruited. Therefore, a strict randomized controlled study involving a large sample should be conducted to evaluate the safety and efficacy of the TTNS device. Second, the urodynamic examination was performed before and during the stimulation but there was no urodynamic evaluation at the end of the treatment period. Third, there was a lack of blinded approaches with the study design. Fourth, long-time follow-up beyond a 4-weeks period should be done in the future.

## Conclusion

This wearable TTNS device demonstrated preliminary feasibility in managing OAB. However, more randomized controlled studies with larger samples are required to substantiate these preliminary findings.

## Data Availability Statement

The original contributions presented in the study are included in the article/supplementary material, further inquiries can be directed to the corresponding authors.

## Ethics Statement

The studies involving human participants were reviewed and approved by Ethics Committee of Beijing Boai Hospital Drug Clinical Trial Organization. The patients/participants provided their written informed consent to participate in this study.

## Author Contributions

LL and XiL designed the study and participated in critical revisions of important knowledge content. XuL and XiL performed the study. HZ and ZZ helped to perform the study. XuL wrote the manuscript. All authors have finally approved the version to be released, agree to be responsible for all aspects of the work, and read and approved the final manuscript.

## Funding

This study was funded by National Key R&D Program of China (2018YFC2002203). The funders had no role in study design, data collection and analysis, decision to publish, or preparation of the manuscript.

## Conflict of Interest

The authors declare that the research was conducted in the absence of any commercial or financial relationships that could be construed as a potential conflict of interest. The handling editor [PZ] declared a shared affiliation with the authors [XuL, XiL, ZZ, LL] at the time of review.

## Publisher's Note

All claims expressed in this article are solely those of the authors and do not necessarily represent those of their affiliated organizations, or those of the publisher, the editors and the reviewers. Any product that may be evaluated in this article, or claim that may be made by its manufacturer, is not guaranteed or endorsed by the publisher.
